# A Castration-Resistant Prostate Tumor With Unusual Anatomy Treated Using Daily Online Adaptive Radiotherapy

**DOI:** 10.7759/cureus.72879

**Published:** 2024-11-02

**Authors:** Mangesh Patil, Harjot Kaur Bajwa, Abhinav Puppalwar, Rajesh Natte, Naveen Mahadevan, Satyam Satyarth, Uday Chandankhede, Suresh Chaudhari, Sushil Beriwal

**Affiliations:** 1 Radiation Oncology, American Oncology Institute, Nagpur, IND; 2 Radiation Oncology, American Oncology Institute, Hyderabad, IND; 3 Medical Physics, American Oncology Institute, Hyderabad, IND; 4 Medical Oncology, American Oncology Institute, Nagpur, IND; 5 Urology, American Oncology Institute, Nagpur, IND; 6 Radiation Oncology, Allegheny Health Network Cancer Institute, Pittsburgh, USA; 7 Medical Affairs, Varian Medical Systems, Inc., Palo Alto, USA

**Keywords:** castrate-resistant prostate cancer, cbct-based oart, daily online adaptive radiotherapy (doart), ethos, igrt, prostate cancer

## Abstract

Daily online adaptive radiotherapy (OART) is useful in radiotherapy of prostate cancer to reduce doses to the rectum and bladder which pose a challenge because of daily variation in shape and size. It also helps to reduce target margins while still maintaining target coverage. We present a case of prostate cancer resistant to androgen deprivation therapy and systemic therapy which was difficult to treat with definitive radiotherapy because of the unusual anatomical shape of the tumor impinging into the rectum. Cone beam computed tomography-based daily OART helped in adapting the dose to changes in the prostate volume during the course of radiation and helped reduce the dose to the rectum while maintaining coverage of the target volume. We could achieve excellent biochemical and radiological responses without significant toxicity.

## Introduction

Online adaptive radiotherapy (OART) can potentially reduce the effect of daily treatment variability, which may be in the form of target position or organ at risk filling. It ensures accurate treatment delivery and allows us to improve the therapeutic ratio while safely reducing the treatment margins [1-3]. However, adaptive radiotherapy has yet to be widely adopted across institutions despite its advantages, as it is technically and logistically challenging.

Variable shape and filling of the rectum due to the presence of gas or fecal matter may change the position of the prostate daily. Variable bladder filling and changes in bowel position and shape are also challenges during radiotherapy of prostate cancer. This results in different target coverage and doses to organs at risk (OARs) daily. OART for prostate cancer addresses these challenges, resulting in better target coverage and a reduction in doses of OARs. The introduction of artificial intelligence (AI)-based calculation engines such as Ethos (Varian Medical Systems, Palo Alto, CA, USA) has enabled daily OART to be performed within a short time frame [4-6]. In this workflow, the patient's anatomy is imaged for each fraction, and specific structures named "influencers" are initially auto-contoured using AI. This is followed by creating targets and OARs on the Ethos cone beam computed tomography (CBCT) and editing by the clinician if necessary. Thus, it provides an opportunity to modify targets and OARs on a daily basis, adapting them to daily variable anatomy. Once the clinical target volume (CTV) has been approved, the predefined CTV-to-planning target volume (PTV) margin is applied to create the target. A new treatment plan is generated daily based on daily CBCT images using the clinical goals prescribed during initial planning. The original treatment plan is known as the reference plan. The reference plan, which is regenerated according to daily CBCT anatomy, is labeled as the scheduled plan, whereas the optimized plan created on daily CBCT is called the adaptive plan.

This case report emphasizes the importance of daily online adaptive planning in a patient with prostate cancer with unusual target anatomy in close proximity to the rectum. In this setting, we have reported the dosimetric advantage of adaptive planning, clinical response, and acute toxicity.

## Case presentation

A 74-year-old male patient presented with complaints of increased frequency of urination of two months duration. He also complained about the urgency of micturition and a weak stream of urination. The American Urological Association (AUA) urinary symptom score was 14/35. The serum total prostate-specific antigen (PSA) level at presentation was 73.9 ng/ml (reference range: 0-4 ng/ml). Transrectal ultrasound-guided biopsy was done, and samples were taken from 14 cores. All cores were reported as prostatic acinar adenocarcinoma except one from the right lateral apex of the prostate gland. Gleason's score was 5 + 5 = 10 (prognostic Grade group 5). Perineural invasion was present in four cores. The percentage of the tumor volume ranged from 5% to 90%. Lymphovascular invasion was identified in one core from the left lateral apex. In immunohistochemistry studies, the tumor cells were positive for markers NKX3.1 and AR and negative for synaptophysin.

**Figure 1:**
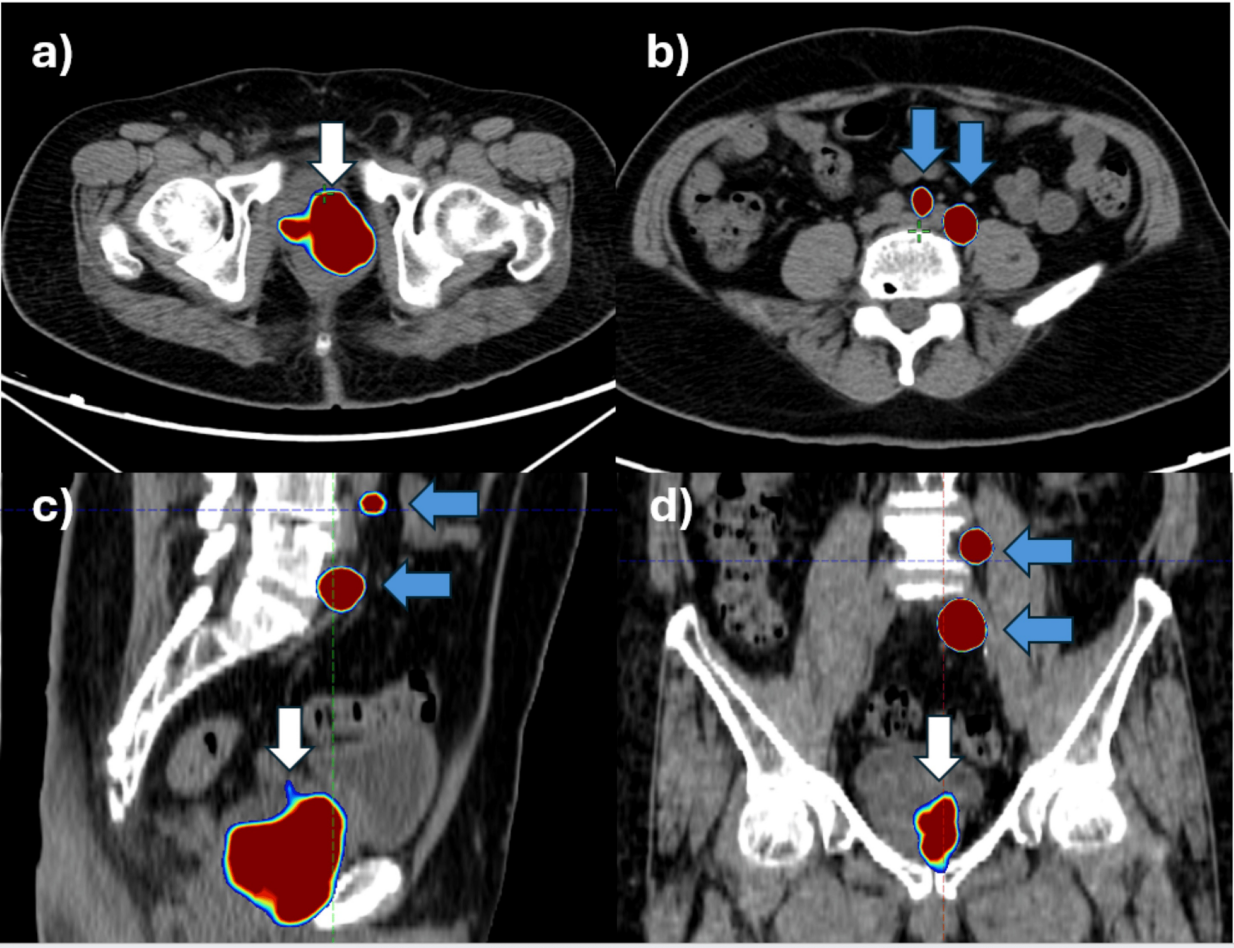
PSMA PETCT shows increased uptake in the prostate (white arrow) and pelvic lymph nodes (blue arrow) in (a) axial, (b) axial, (c) sagittal, and (d) coronal sections PSMA PETCT: prostate-specific membrane antigen positron emission tomography-computed tomography

Whole body prostate-specific membrane antigen positron emission tomography-computed tomography (PSMA PETCT) imaging revealed PSMA expressing mixed density irregular soft tissue lesion in the peripheral zone of the left lobe of the prostate gland, which contiguously involved the paramedian peripheral zone of the right lobe and central/ transitional zones of the left lobe. The lesion measured 6.4, 5.8, and 6.3 centimeters in transverse, anteroposterior, and craniocaudal directions, respectively, and had an SUV max value of 22.6 (Figure 1a). Posteriorly, complete obliteration of the left recto prostatic angle was noted with adherence to the anterior wall of the rectum. The lesion had extra-prostatic extension into seminal vesicles. The urinary bladder was uninvolved by the tumor. PSMA avid right external iliac, bilateral internal iliac, and left common iliac nodes were present (Figure 1). The largest node measured 2.6x1.6 centimeters (SUV max- 49.1) in the left common iliac region. No distant metastatic lesions were identified. The patient received androgen deprivation therapy (ADT) in the form of an Injection of Leuprolide 11.25 mg via the subcutaneous route. The patient started taking abiraterone tablets after the first dose of leuprolide, as advised by the treating urosurgeon.

**Figure 2:**
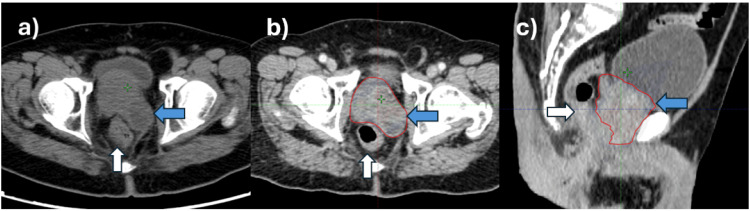
CT image (a) axial section shows a tumor in the prostate with a cystic component (blue arrow) on the left side impinging on the rectum (white arrow), (b) target volume (blue arrow) encompassing the entire prostate and cystic component on the left side in the axial section, and (c) sagittal section CT: computed tomography

The patient came to our institution just after starting ADT for an opinion regarding radiotherapy. He was stratified as very high risk and staged as cT3bcN1 M1a (stage IV B) as per TNM staging American Joint Committee on Cancer (AJCC) Version 8. The tumor in the prostate had a cystic component on the left side measuring 4 x 4 centimeters, which extended from the left anterior portion of the prostate to almost up to the posterior wall of the rectum. The cystic component covered the space adjacent to the left lateral wall of the rectum, pushing the rectum laterally to the right side (Figure 2a). It was anticipated that the close proximity of the tumor to the rectum would not allow safe definitive radiotherapy (Figures 2b, 2c). It was decided in the tumor board to wait for response post-leuprolide and abiraterone and then reassess for curative radiotherapy.

Serum total PSA levels after two doses of ADT were 14 ng/ml. The patient was screened by computed tomography to check for response in the primary lesion, which was stable. Serum testosterone levels were 60 ng/dl, suggesting non-castrate testosterone levels. The tumor was challenged with four cycles of docetaxel 110 mg (75 mg per square meter of BSA) intravenously once every 21 days. ADT was revised to Injection Degarelix 240 mg initial dose and 80 mg monthly dose every month. Abiraterone tablets were stopped. Serum total PSA levels after four cycles of docetaxel were 12.7 ng/ml.

In view of the poor biochemical response (serum PSA 12.7 ng/ml), it was decided to use definitive doses of radiotherapy as the cancer was still confined to the pelvis region only. Delivering a curative dose of radiotherapy was a big challenge because of the close proximity of the tumor to the left lateral wall of the rectum and the large tumor size. PTV margin, which usually overlaps only with the anterior wall of the rectum, would, in this case, overlap with the anterior and left lateral wall of the rectum. This would lead to high-dose spillage in the rectum, significantly increasing the risk of rectal toxicity. Daily changes in prostate and rectal volume would bring about larger variations in dose deposition in the rectum, considering the increased area of overlap of PTV with the rectum.

The option of daily OART was proposed to reduce the risk of toxicity to the rectum and deliver a definitive radiation dose to the primary prostate lesion. The patient was referred to our affiliated center in a different city to avail of the OART facility. PSMA PETCT was done for re-staging before the initiation of curative radiotherapy. PSMA PETCT revealed interval resolution of the previously seen PSMA uptake in the prostate gland. However, there was an increase in PSMA concentration within the extra-prostatic solid cystic component arising from the left lateral peripheral zone of the prostate (size 3.7 x 3.2 cm, SUV max 57.99, previously 17.25). There was interval regression in size and PSMA concentration within the bilateral common, internal, and external iliac lymph nodes (sub-centimeter size, SUV max 17.25 vs 43.22 previously). No other metastatic lesions were present.

Pre-planning

The patient was counseled regarding the treatment planning and advised to follow a bladder protocol during the simulation and every day before treatment. The bladder protocol advised was to drink 500ml of water 30 minutes before simulation and treatment. The patient was advised to take laxatives every night before radiation and come for treatment in the morning to maintain an empty rectum. The patient was simulated with a full-body vacuum cushion and knee rest. Planning CT images of 1.25mm slice thickness were acquired.

**Figure 3:**
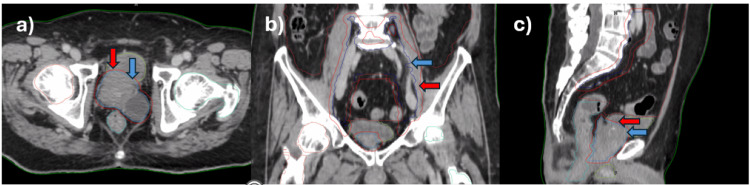
Planning CT images showing target and OAR contours in (a) axial, (b) coronal, and (c) sagittal sections. In pane a, the blue contour is CTVp (blue arrow), and the red contour is PTVp (red arrow). In pane b, the blue contour is CTVn (blue arrow), and the red contour is PTVn (red arrow). In pane c, CTVp (blue arrow) and PTVp are visualized CT: computed tomography, OAR: organs at risks, CTVp: clinical target volume of the primary tumor, PTVp: primary planning target volume, CTVn: clinical target volume of nodes, PTVn: planning target volume nodes

Magnetic resonance imaging (MRI) was also performed, and T2-weighted images were used for contouring after fusion with planning CT images. Images from pre-simulation PSMA PET were also uploaded in the patient study set to aid in contouring and planning, as the prostatic cyst had intense PSMA PET uptake along with the entire prostate and proximal seminal vesicles. Target and OARs were contoured on the Ethos planning system by a qualified radiation oncologist as per the contouring guidelines (Figures 3a-3c). The primary gross tumor volume (GTVp) included the entire prostate, prostatic cyst, and base of the seminal vesicles (SV). The clinical target volume of the primary tumor (CTVp) comprised GTVp and the entire SV. The clinical target volume of nodes (CTVn) included the entire common iliac, internal and external iliac, and pre-sacral lymph nodes. The gross nodes were contoured on CT slices using information from the PSMA PET scan as gross tumor volume of nodes (GTVn). The primary planning target volume (PTVp) was directly created as a derived structure in the Ethos planning system by giving a margin to CTVp (5mm all around except 3mm posteriorly). The planning target volume nodes (PTVn) intended to receive 62.5 Gy dose in 25 fractions were created from GTVn as a derived structure by giving a 3mm margin to GTVn. CTVn was given a 5mm margin to generate PTV50 (planning target volume receiving 50 Gy in 25 fractions). The OARs comprise the bladder, rectum, bowel, penile bulb, and femoral heads.

The planning aim was to deliver a dose of 68Gy in 25 fractions over five weeks to the primary, 62.5Gy in 25 fractions to the gross nodes, and 50Gy in 25 fractions to the prophylactic nodal chain using the simultaneous integrated boost technique. The clinical goals were fed into the Ethos treatment planning system for pre-planning and optimization. The clinical goals are described in Table 1. The Ethos system generates a dose preview for pre-plan optimization based on the clinical goals defined by the user. After assigning priority to clinical dose parameters, a final plan was generated. Dose distribution for nine fields intensity-modulated radiotherapy (IMRT), 12 fields IMRT, and volumetric modulated arc therapy plans were compared, and the nine fields IMRT plan was chosen for final treatment delivery and daily online adaptation.

**Table 1:** Clinical goals for Ethos adaptive planning and reference plan doses CTV: clinical target volume, PTV: planning target volume, cc: cubic centimeters, CTV68: clinical target volume receiving 68Gy in 25 fractions, CTV50: clinical target volume receiving 50Gy in 25 fractions, PTV68: planning target volume receiving 68Gy in 25 fractions, PTVN62.5: planning target volume of nodes receiving 62.5Gy in 25 fractions, PTV50: planning target volume receiving 50Gy in 25 fractions, D0.03cc: dose to 0.03 cc volume, Dmean: mean dose, V100%: volume receiving 100% of the prescribed dose, V95%: volume receiving 95% of the prescribed dose, V105%: volume receiving 105% of the prescribed dose, V65Gy: volume receiving 65Gy, V60Gy: volume receiving 60Gy, V50Gy: volume receiving 50Gy, V40Gy: volume receiving 40Gy, V30Gy: volume receiving 30Gy, Ethos: Varian Medical Systems, Palo Alto, CA, USA

Structure	Clinical goals (objectives)		Reference plan doses
CTV	CTV68	V 100% ≥ 95%	100%
	CTV50	V 100% ≥ 95%	99.9%
PTV	PTV68	V 100% ≥ 95%	95.4%
		V 95% ≥ 99%	99.4%
		D 0.03 cc ≤ 107%	109.5%
		V 105% ≤ 5%	7.5%
	PTVN62.5	V 105% ≤ 5%	0%
		D 0.03 cc ≤ 107%	104.2%
		V 95% ≥ 95%	95.8%
	PTV50	V 100% ≥ 97%	98.6%
		V 105% ≤ 5%	9.5%
		D 0.03 cc ≤ 108%	125%
Bladder		V 65 Gy < 4%	8.9%
		V 60 Gy ≤ 11%	14.4%
		V 50 Gy ≤ 25%	26.7%
		V 40 Gy ≤ 42%	45.8%
		V 30 Gy ≤ 58%	71.2%
Rectum		V 65 Gy < 3%	4.2%
		V 60 Gy < 10%	8.2%
		V 50 Gy ≤ 25%	18.1%
		V 40 Gy ≤ 38%	37.2%
		V 30 Gy ≤ 68%	68.1%
Bowel bag		V 50 Gy < 70 cc	165.85 cc
Femur head and neck left		V 40 Gy ≤ 5%	0.1%
Femur head and neck right		V 40 Gy ≤ 5%	0.4%
Penile bulb		D mean ≤ 15 Gy	12.05Gy

Daily online adaptive treatment

The patient was treated in the morning daily following the bladder protocol described above. The radiation oncologist was present daily for contour editing and plan approval. After positioning, a CBCT was acquired using the pelvis large protocol. An iterative CBCT was reconstructed using Ethos software and approved for contouring. The prostate, seminal vesicles, bladder, rectum, and bowel were auto-contoured as influencer structures by Ethos. These structures were modified if necessary or accepted to proceed to target generation. Once the targets were generated, they were compared with the planning CT/MRI images and edited using editing tools. The GTV encompassed the prostate, seminal vesicles, and the cystic nodule, which was in close proximity to the rectum. The OARs (penile bulb and femur heads) were contoured using deformable image registration between planning CT and CBCT. Once the editing was complete, the structures were approved, and a synthetic CT was generated for online dose optimization and calculation. Based on the clinical goals prescribed at the time of initial planning, the Ethos system generated two IMRT plans (nine fields) corresponding to the anatomy of the day, called a scheduled plan and an adaptive plan. The scheduled plan depicted the dose delivered to the target and OARs based on the original pre-plan but recalculated on present-day CBCT. Based on current anatomy, the adaptive plan depicted the dose delivered to the target and OARs after re-optimization.

The dose comparison between the adaptive plan and the scheduled plan is depicted in Tables 2-4. The radiation oncologist approved the final plan daily based on target coverage and OAR sparing. After approval and before treatment delivery, another CBCT was acquired for position verification and offline review. Couch shifts were applied, if necessary, and the selected plan was delivered. The median time of the entire treatment session, including the adaptive process, from the first CBCT acquisition to treatment delivery was 30 minutes (range 25-45 minutes). The patient was treated using the adaptive plan in all fractions. 

**Table 2:** Comparison of daily scheduled vs adaptive plan for target coverage CTV: clinical target volume, PTV: planning target volume, cc: cubic centimeters, CTV68: clinical target volume receiving 68Gy in 25 fractions, CTV50: clinical target volume receiving 50Gy in 25 fractions, PTV68: planning target volume receiving 68Gy in 25 fractions, PTVN62.5: planning target volume of nodes receiving 62.5Gy in 25 fractions, PTV50: planning target volume receiving 50Gy in 25 fractions, D0.03cc: dose to 0.03 cc volume, V100%: volume receiving 100% of the prescribed dose, V95%: volume receiving 95% of the prescribed dose, V105%: volume receiving 100% of the prescribed dose

Structure	Objective		Scheduled plan (average%)	Adaptive plan (average%)
CTV	CTV68	V 100% ≥ 95%	94%	98.8%
	CTV50	V 100% ≥ 95%	97%	100%
PTV	PTV68	V 100% ≥ 95%	88%	90%
		V 95% ≥ 99%	90%	97.6%
		D 0.03 cc ≤ 107%	108%	104.3%
		V 105% ≤ 5%	4%	0%
	PTV62.5	V 95% ≥ 95%	78.79%	97.46%
		V 105% ≤ 5%	4%	4%
		D 0.03 cc ≤ 108%	124%	116%
	PTV50	V 100% ≥ 97%	88%	96.2%
		V 105% ≤ 5%	4%	4%
		D 0.03 cc ≤ 108%	124%	116%

**Table 3:** Rectum dose comparison between the scheduled plan and adaptive plan V65Gy: volume receiving 65Gy, V60Gy: volume receiving 60Gy, V50Gy: volume receiving 50Gy, V40Gy: volume receiving 40Gy, V30Gy: volume receiving 30Gy

Rectum dose constraints	Scheduled plan dose (average%)	Adaptive plan dose (average %)
V65 Gy < 3%	14.85%	3.09%
V60 Gy < 10%	21.41%	8.1%
V50 Gy < 25%	35.89%	19.98%
V40 Gy < 38%	57.26%	36.45%
V30 Gy < 68%	91.97%	70.67%

**Table 4:** Bladder dose comparison between the scheduled plan and adaptive plan V65Gy: volume receiving 65Gy, V60Gy: volume receiving 60Gy, V50Gy: volume receiving 50Gy, V40Gy: volume receiving 40Gy, V30Gy: volume receiving 30Gy

Bladder dose constraints	Scheduled plan dose (average %)	Adaptive plan dose (average %)
V65 Gy < 4%	17.1%	9.48%
V60 Gy < 11%	21.80%	14.13%
V50 Gy < 25%	32.34%	24.63%
V40 Gy < 42%	49.26%	42.34%
V30 Gy < 58%	74.41%	66.94%

Response of tumor to radiotherapy

**Figure 4:**
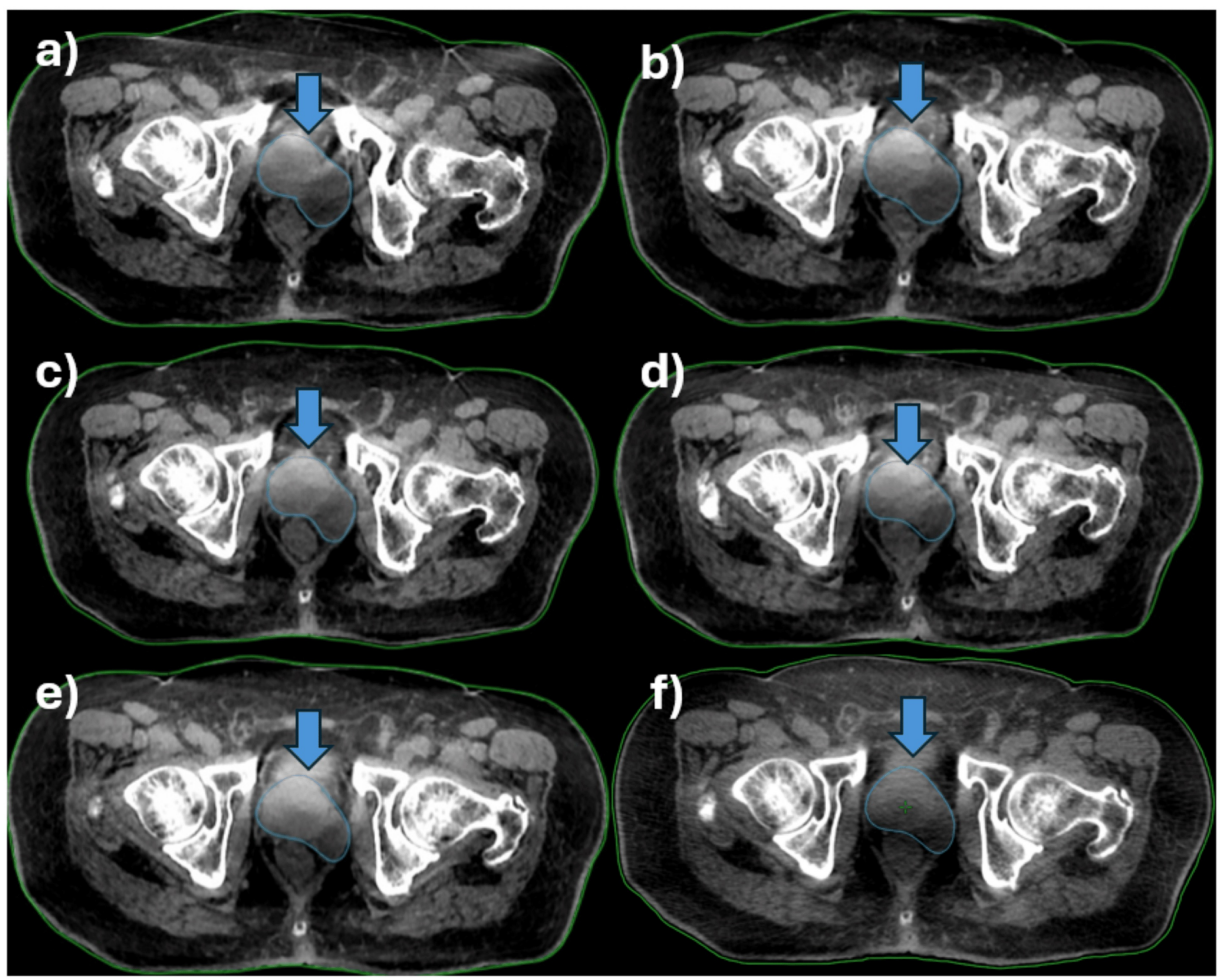
Weekly response of the primary tumor in the prostate during daily OART as seen on CBCT images acquired in a) first week, b) second week, c) third week, d) fourth week, e) fifth week, and f) last day of treatment. CTVp (blue arrow) is contoured using a blue outline. OART: online adaptive radiotherapy, CBCT: cone beam computed tomography, CTVp: clinical target volume of the primary tumor

**Figure 5:**
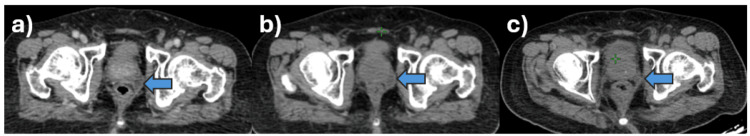
Response in the primary tumor in the prostate (blue arrow) at (a) two weeks, (b) eight weeks, and (c) four months post-OART OART: online adaptive radiotherapy

The target's response was recorded weekly to check for tumor regression (Figure 4). Two weeks after completion of radiotherapy, the serum PSA levels dropped to 0.15 ng/ml; at eight weeks, it was 0.13 ng/ml. The cystic component of the lesion and the entire prostatic tumor significantly reduced in size, as seen on CT two weeks after radiotherapy completion (Figure 5a). This morphologic response kept on increasing till eight weeks post-RT completion (Figure 5b). A complete response in all lymph nodes was evident in two weeks. Serum PSA levels after four months of completion of radiotherapy were 0.02 ng/ml. PSMA PETCT at four months demonstrated a complete metabolic response and significant shrinkage of the cystic component (Figure 5c).

Toxicity

The patient was assessed for toxicity every week during radiotherapy and at each visit on follow-up. Acute toxicity was graded according to the radiation therapy oncology group (RTOG) toxicity grading (Table 5). The patient reported grade 1 acute gastrointestinal (GI) and grade 1 acute genitourinary (GU) toxicity during radiotherapy. Both the toxicities were resolved on follow-up at three months. The patient did not report any Grade 3 or 4 toxicity during treatment or till four months post-OART.

**Table 5:** Acute toxicity assessment during OART and on follow-up (RTOG grading) GI: gastrointestinal, GU: genitourinary, OART: online adaptive radiotherapy, RTOG: Radiation Therapy and Oncology Group

Acute toxicity	Week 1	Week 2	Week 3	Week 4	Week 5	Post RT-2 Weeks	Post RT-8 Weeks	Post RT-3 months
Lower GI toxicity	Grade 0	Grade 0	Grade 0	Grade 1	Grade 1	Grade 0	Grade 0	Grade 0
GU toxicity	Grade 0	Grade 0	Grade 1	Grade 1	Grade 1	Grade 1	Grade 1	Grade 0

## Discussion

OART is used in prostate cancer for its capacity to account for inter-fractional variations in volumes and shapes of the bladder and rectum due to the variable filling of these organs. Delivering the dose calculated based on the adaptive plan optimized using the anatomy of the bladder and rectum on the day of treatment while the patient is in treatment position on the couch reduces the dose to the bladder and rectum [5,7]. There is limited data regarding the outcomes of daily OART in prostate cancer patients with variable anatomy. Most prostate cancer patients have difficulty in maintaining a full bladder. Even with a bladder protocol, there can be a significant variation in bladder filling over the entire course of treatment. Similarly, it is difficult to maintain an empty rectum as patients receive treatment as per machine availability. In our patient, prostate and seminal vesicles did not require editing or required minor editing (three or fewer CT slices) over the entire 25 fractions. The bladder and rectum required minor edits, and the bowels required major edits ( more than three CT slices ) during the course of treatment. This is congruent with the study published by Byrne et al., where 11% of the influencer contours required no change, and 81% required minor edits [5]. The major bowel editing in our patient correlates with the fact that he could tolerate only a moderate bladder filling and had a variable bladder volume at each fraction.

**Figure 6:**
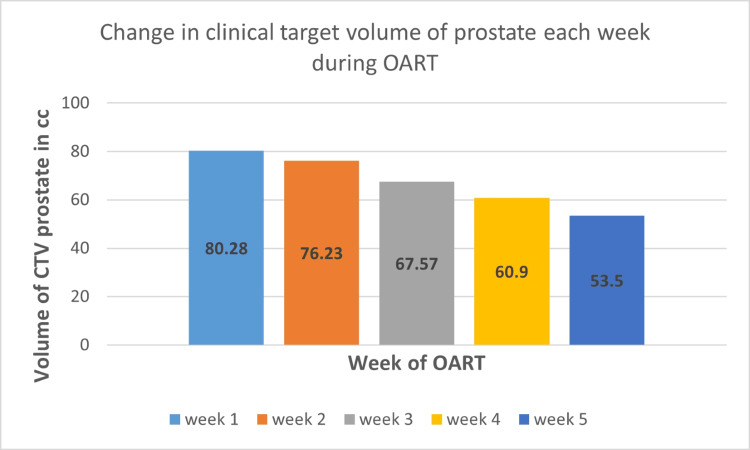
Change in the clinical target volume of the prostate each week during OART Values on the Y axis indicate volume in cubic centimeters. Bars on the X axis indicate the volume of CTV prostate each week during OART. CTV: clinical target volume, cc: cubic centimeters, OART: online adaptive radiotherapy

**Figure 7:**
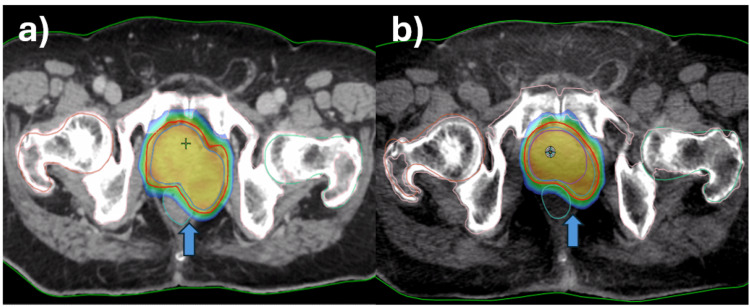
Radiotherapy dose distribution (blue arrow) in (a) planning CT vs (b) last week CBCT CT: computed tomography, CBCT: cone beam computed tomography

This patient was unique as he had a malignant prostatic cyst that protruded toward the lateral border of the rectum and almost reached up to the posterior border of the rectum (Figures 2a-2c). There was a significant risk of over-dosing the rectum or underdosing the target due to variable rectal filling or gaseous distension. In this case, the main advantage of daily OART was its capacity to reoptimize target coverage based on the anticipated decrease in the volume of the cystic tumor as a response to radiotherapy. The prostatic cyst shrunk significantly (34%) over the course of treatment, which is very unusual for commonly treated prostate cancer (Figure 6). Thus, with daily online adaptive contouring and planning, we could reduce the target volume, which further led to reduced rectal doses and improved target coverage. Due to the unusual location of the cyst, this decrease in volume created a space between the CTV and the rectum, thereby reducing the overlap of the rectum with the PTV, mainly on the left lateral side. The dose comparison between the initial planned CT and Ethos CBCT acquired during the last week is depicted in Figure 7.

There is limited data regarding the benefit of OART in prostate cancer patients with lymph node involvement. Christiansen et al. analyzed the doses to the organs at risk in high-risk prostate cancer patients, receiving 78Gy to the prostate and 56Gy to the elective pelvic lymph nodes in 39 fractions using MRI-guided adaptive radiotherapy [1]. They concluded that online adaptive radiotherapy yielded statistically significant lower doses for the bladder wall, rectum, and peritoneal cavity compared to the standard RT. In our patient, we treated the prostate and lymph nodes with a boost to the gross nodes. The adaptive plan was selected over the scheduled plan for all fractions as it was superior in terms of either target coverage and/or OAR sparing. Upon comparison of the cumulative doses delivered, the rectum received less dose across all volumes with the adaptive plan as compared to the scheduled plan (Table 3). The rectal constraint of volume receiving 65 Gy (V65Gy) < 3% was achieved in the adaptive plan across all fractions, whereas in the scheduled plan, the V65Gy constraint exceeded the limit (cumulative V65= 17%). While maintaining coverage of regressing prostate volume, the V65Gy constraint of the rectum was reduced by more than fivefold (17% vs 3.2%). The primary target and nodal coverage were superior in the adaptive plan compared to the scheduled plan. The cumulative bowel volume receiving 50Gy was 130cc with an adaptive plan compared to 165cc with a scheduled plan. The penile bulb achieved a mean dose of 10Gy with an adaptive plan compared to 12Gy with the scheduled plan.

Another approach for performing OART includes the use of MRI guidance for daily target and OAR visualization and treatment [1,3,8]. Even though MRI provides better soft tissue contrast and intra-fraction monitoring, the entire adaptive workflow process takes around 45 minutes for each patient. Our adaptive workflow took around 20 minutes daily. The target and organs at risk were well visualized. The pre-treatment CBCT taken after adaptive planning showed minimal or no shift with mild variation in bladder filling at the superior aspect. The part of the bladder close to the target did not vary in position.

Teunissen et al. analyzed the 12-month outcomes of 425 patients with localized prostate cancer treated with MRI-guided radiotherapy. At three months post-treatment, the majority of the toxicities were grade 1, followed by grade 2 toxicities [3]. The acute toxicities in this patient were limited to Grade 1 toxicity only. There was no Grade 3 or 4 acute toxicity. Good target coverage and reduced OAR doses in OART manifested into a complete metabolic response and excellent biochemical and morphological response with acceptable toxicities in this patient.

## Conclusions

This is a very unusual case of prostate cancer, which benefited from daily OART because of significant changes in the prostate volume from tumor regression. Daily OART made it possible to deliver curative doses of radiation when the volume and unusual shape of the tumor and change in the tumor volume during radiotherapy posed a challenge to respect OAR rectum tolerance doses. Physicians are more likely to choose adaptive plans on most days because of improved target coverage and reduced OAR dose.
